# LRP1 is a neuronal receptor for α-synuclein uptake and spread

**DOI:** 10.1186/s13024-022-00560-w

**Published:** 2022-09-02

**Authors:** Kai Chen, Yuka A. Martens, Axel Meneses, Daniel H. Ryu, Wenyan Lu, Ana Caroline Raulin, Fuyao Li, Jing Zhao, Yixing Chen, Yunjung Jin, Cynthia Linares, Marshall Goodwin, Yonghe Li, Chia-Chen Liu, Takahisa Kanekiyo, David M. Holtzman, Todd E. Golde, Guojun Bu, Na Zhao

**Affiliations:** 1 Department of Neuroscience, Mayo Clinic, 4500 San Pablo Road, Jacksonville, FL 32224 USA; 2 Departments of Neuroscience and Neurology, University of Florida, Gainesville, FL 32611 USA; 3 Department of Neurology, Hope Center for Neurological Disorders, Knight Alzheimer’s Disease Research Center, Washington University School of Medicine, St. Louis, MO 63110 USA

**Keywords:** Low-density lipoprotein receptor-related protein 1, α-Synuclein, Human induced pluripotent stem cells, Parkinson’s disease, Lewy body dementia

## Abstract

**Background:**

The aggregation and spread of α-synuclein (α-Syn) protein and related neuronal toxicity are the key pathological features of Parkinson’s disease (PD) and Lewy body dementia (LBD). Studies have shown that pathological species of α-Syn and tau can spread in a prion-like manner between neurons, although these two proteins have distinct pathological roles and contribute to different neurodegenerative diseases. It is reported that the low-density lipoprotein receptor-related protein 1 (LRP1) regulates the spread of tau proteins; however, the molecular regulatory mechanisms of α-Syn uptake and spread, and whether it is also regulated by LRP1, remain poorly understood.

**Methods:**

We established *LRP1* knockout (*LRP1*-KO) human induced pluripotent stem cells (iPSCs) isogenic lines using a CRISPR/Cas9 strategy and generated iPSC-derived neurons (iPSNs) to test the role of LRP1 in α-Syn uptake. We treated the iPSNs with fluorescently labeled α-Syn protein and measured the internalization of α-Syn using flow cytometry. Three forms of α-Syn species were tested: monomers, oligomers, and pre-formed fibrils (PFFs). To examine whether the lysine residues of α-Syn are involved in LRP1-mediated uptake, we capped the amines of lysines on α-Syn with sulfo-NHS acetate and then measured the internalization. We also tested whether the N-terminus of α-Syn is critical for LRP1-mediated internalization. Lastly, we investigated the role of Lrp1 in regulating α-Syn spread with a neuronal *Lrp1* conditional knockout (*Lrp1*-nKO) mouse model. We generated adeno-associated viruses (AAVs) that allowed for distinguishing the α-Syn expression versus spread and injected them into the hippocampus of six-month-old *Lrp1*-nKO mice and the littermate wild type (WT) controls. The spread of α-Syn was evaluated three months after the injection.

**Results:**

We found that the uptake of both monomeric and oligomeric α-Syn was significantly reduced in iPSNs with *LRP1*-KO compared with the WT controls. The uptake of α-Syn PFFs was also inhibited in *LRP1*-KO iPSNs, albeit to a much lesser extent compared to α-Syn monomers and oligomers. The blocking of lysine residues on α-Syn effectively decreased the uptake of α-Syn in iPSNs and the N-terminus of α-Syn was critical for LRP1-mediated α-Syn uptake. Finally, in the *Lrp1*-nKO mice, the spread of α-Syn was significantly reduced compared with the WT littermates.

**Conclusions:**

We identified LRP1 as a key regulator of α-Syn neuronal uptake, as well as an important mediator of α-Syn spread in the brain. This study provides new knowledge on the physiological and pathological role of LRP1 in α-Syn trafficking and pathology, offering insight for the treatment of synucleinopathies.

**Supplementary Information:**

The online version contains supplementary material available at 10.1186/s13024-022-00560-w.

## Background

The α-synuclein (α-Syn) is a 140-residue protein encoded by the *SNCA* gene that is abundantly expressed in the nervous system, predominantly in neurons [1, 2]. The α-Syn protein is enriched at presynaptic terminals where it functions to sustain normal soluble *N*-ethylmaleimide–sensitive factor attachment protein receptor (SNARE)-complex structure [3], thus playing important roles in synaptic processes, such as vesicle trafficking/recycling and neurotransmitter release [4–7]. It has been shown that α-Syn protein can be secreted into the extracellular space including brain interstitial fluid (ISF), cerebrospinal fluid (CSF), and plasma, although the exact mechanism of α-Syn secretion is not well understood [8–11]. Importantly, these secreted soluble α-Syn can be taken up by different cells. Particularly, the oligomeric α-Syn species appear to be prone to uptake and spread from cell-to-cell in a prion-like manner, leading to the templated misfolding of the native forms of α-Syn and formation of α-Syn aggregates throughout the brain, which are the defining pathological features of Parkinson’s disease (PD) and dementia with Lewy body (DLB) [12–19]. However, the underlying cellular mechanisms of α-Syn uptake and spread remains poorly understood.

The low-density lipoprotein receptor (LDLR)-related protein 1 (LRP1) is a transmembrane receptor that belongs to the LDLR family [20]. LRP1 mediates and regulates the endocytosis of  > 30 ligands, including apolipoprotein E (APOE) and amyloid-β (Aβ) [21–27], thus playing a critical role in the pathogenesis of Alzheimer's disease (AD) [28]. A recent study also identified LRP1 as a key regulator for the uptake and spread of microtubule-associated protein tau [29]. Although tau and α-Syn have key differences in their pathological roles, they do share similarity in cell-to-cell transmission and pathological spread [30]. Tau interacts with LRP1 through lysine residues in the microtubule-binding repeat region [29]. Notably, α-Syn has a similar high content (10%) of lysines as tau. These common characteristics between tau and α-Syn suggest that their uptake and spread might be regulated by the common multiple-functional receptor LRP1.

Here in this study, we tested the role of LRP1 in mediating α-Syn uptake and spread using human induced pluripotent stem cells (iPSCs)-derived neurons (iPSNs) and a conditional transgenic mouse model. In iPSNs, the monomeric and oligomeric α-Syn uptake was significantly reduced when *LRP1* gene was knocked out (*LRP1*-KO) using the CRISPR/Cas9 strategy. We further confirmed that the α-Syn N-terminal domain interacted with LRP1 through lysine residues. Lastly, we constructed adeno-associated viruses (AAVs) to express human α-Syn in neurons and visualized the α-Syn spread in mouse brains. We found that the spreading of α-Syn was reduced in mouse brain with neuronal *Lrp1* deletion. Altogether, our results suggest that LRP1 is a key regulator for neuronal α-Syn uptake and spread, providing insight into the mechanisms of α-Syn pathogenesis.

## Methods

### Generation of *LRP1*-KO iPSCs

Fibroblasts from a healthy individual (female; 83-year-old; *APOE3/3* genotype) obtained from Mayo Clinic Neuroregeneration Lab were reprogrammed into iPSC clones (MC0192). Clone #4 of MC0192 was used as parental iPSCs for gene editing. Isogenic *LRP1*-KO iPSC lines were obtained via a CRISPR/Cas9 gene editing method by ALSTEM Inc. Three gRNA/Cas9 constructs for human *LRP1* were designed to target exon 6 of the *LRP1* gene [*LRP1* gRNA1: ATCTTGGCCACGTACCTGAG; *LRP1* gRNA2: ATGCCAACGAGACCGTATGC; and *LRP1* gRNA3: TGACTCACGGTGCAGACTGA] (Fig. S1a). The parental iPSCs were cultured in complete mTeSR^TM^1 media plus Pen/Strep antibiotics at 37 °C with 5% CO2. About 3 × 10^5^ cells were transfected with 1.5 μg of each gRNA/Cas9 plasmids by Invitrogen Neon transfection system. After transfection, cell lysate was used to examine the knockout efficiency by PCR (primers: *LRP1-F* CACGGACTCTTCTCTTCCCC; *LRP1-R* TCCCGGCCTCTGTTCAAGAT).

Single cells were plated in multiple 96-well plates and cultured for 14 days before expanding to 12-well plates. Genomic DNA was subsequently extracted from single cell clones and used for PCR analysis for identifying knockout clones. Knockout was further confirmed by DNA sequencing.

### Differentiation of human iPSCs into neurons

Human iPSCs were differentiated into neurons as previously described [31, 32]. Briefly, iPSCs were maintained in Matrigel (Corning, cat# 354277)-coated plates using mTeSR™1 medium (Stemcell Technologies, cat# 85850). To initiate neurosphere formation, iPSCs were plated onto AggreWell 800 24-well plates (Stemcell Technologies, cat# 34811) and cultured with neural induction medium (Stemcell Technologies, cat# 08610) in suspension for 5–7 days. Then, neurospheres were seeded onto Matrigel-coated dishes and cultured in neural induction medium for another 5–7 days to induce neural rosette formation. Next, neural rosettes were isolated as a single cell suspension and re-plated onto Matrigel-coated dishes in neural induction medium. To differentiate the cells into neural progenitor cells (NPCs), the medium was replaced to neural progenitor cell medium (Stemcell Technologies, cat# 05834) and cultured for additional 10–14 days. NPCs were amplified and frozen stocks were made for further experiments. To induce neuron differentiation, NPCs were seeded onto Matrigel-coated plates in a neural progenitor cell medium. The following day, the medium was replaced with neuronal differentiation medium, composed of DMEM/F12 and Neurobasal Medium (1:1) supplemented with N2, B27, BDNF (20 ng/mL), GDNF (20 ng/mL), NT3 (10 ng/mL), IGF (10 ng/mL), ascorbic acid (200 μM) (all from Stemcell Technologies) and dbcAMP (100 nM) (Sigma-Aldrich). NPCs were cultured with neuronal differentiation medium for additional 14 days for differentiation to neurons.

### Protein labeling

Commercial recombinant proteins, including human Tau (R&D Systems, SP-495), α-Syn (Proteos, cat# RP-003), oligomeric α-Syn (StressMarq, cat# SPR-484), α-Syn preformed fibrils (PFFs) (StressMarq, cat# SPR-322-C), α-Syn N (1–60) fragment (rPeptide, cat# S-1011–1), and α-Syn ΔN (61–140) fragment (rPeptide, cat# S-1013–1) were labeled with Alexa Fluor® 488 ester (Life Technologies, cat# A10235) according to the manufacturer’s instructions. After labeling, 100 mM glycine was added to quench the reaction and the proteins were subjected to Amicon Ultra-0.5 mL Centrifugal Filters (Millipore, cat# UFC500396 -3 KDa, cat# UFC501096 -10 KDa, and cat# UFC510096 -100 KDa) to remove any unreacted label. The fluorescently labeled α-Syn PFFs were sonicated using a Qsonica Q125 Sonicator at 30% amplitude for 30 cycles (1 s ON, 1 s OFF) before incubating with cells. Lysine capped proteins were prepared with Sulfo-NHS-Acetate (Thermo, cat# 26777) according to the manufacturer’s instructions.

### Transmission electron microscopy

The morphology of α-Syn species was confirmed by negative stain transmission electron microscopy. α-Syn oligomers (StressMarq, cat# SPR-484) and PFFs (StressMarq, cat# SPR-322-C) were prepared at 25 µM in water. Samples (4 μL) were deposited onto 400-mesh carbon-coated grids (Agar Scientific) and incubated for 1 min before blotting the excess solution off. Grids were washed with water and blotted dry prior to negatively staining the samples with 4 μL filtered 0.5% uranyl acetate for 1 min. Grids were then dried with filter paper and left to air-dry for 5 min before storage. Electron micrographs were obtained using a Jeol JEM-1400Flash transmission electron microscope (Jeol) fitted with a Matataki™ 4 M Flash camera Gatan camera at an operating voltage of 80 kV.

### Protein uptake assay

The iPSNs at DIV 14 were plated at 300,000 cells per well in a 12-well plate. The next day, the medium was replaced, and cells were treated with 100 nM of Alexa Fluor 488-labeled monomeric α-Syn, α-Syn N (1–60) fragment, α-Syn ΔN (61–140) fragment, monomeric tau, or 100 nM (monomer equivalent) of labeled oligomeric α-Syn or sonicated α-Syn PFFs for 3 h at 37 °C. The Alexa Fluor 488-labeled transferrin at 300 nM was treated as controls. After the treatment, the cells were washed twice with phosphate-buffered saline (PBS), trypsinized for 5 min at 37 °C, and lifted from the plate. The cells were collected and analyzed using an Attune™ NxT Flow Cytometer (Thermo Fisher). Total of 10,000 events were recorded per sample. Data analysis was performed using FlowJo software. First, cells were gated on forward scatter area/side scatter area (FSC-A/SSC-A); cells were then gated on forward scatter height (FSC-H) versus FSC-A to discriminate doublets; positive cells were determined by gating on a negative (no protein added) population.

Experiments were run in biological duplicates or triplicates and at least three independent experiments were performed. For the receptor-associated protein (RAP) competition experiments, the recombinant RAP protein (in house) was added into the medium at indicated concentrations (1.5625, 3.125, 6.25, 12.5, 25, 50, and 100 nM) at the same time as α-Syn treatment. For α-Syn fragment competition experiments, five-fold molar excessive (500 nM) of non-labeled α-Syn N (1–60) or ΔN (61–140) fragment was added into the medium at the same time as 488-labeled α-Syn (100 nM).

### Cloning and AAV production

The *pAAV-Synapsin-EGFP-Synapsin-mRuby2* plasmid was used as the backbone for generating the *pAAV-Synapsin-EGFP-Synapsin-h-α-Synuclein* construct [33]. The *mRuby2* gene was replaced with the full-length human α-Syn cDNA using *EcoRV* and *XhoI* restriction sites. The construct was then packaged into AAV2/8 as follows. HEK293T cells (ATCC cat# CRL3216) were cultured to ~ 70% confluency in two Cellstacks (Corning cat# 3269) per construct and transfected using PEI 25 k MW (Polysciences cat# 23966–1) for 3 days. The cells were then harvested via shaking and centrifugation until cell pellet was formed. The pellet was then digested with a final concentration of 50 U/mL of Benzonase (Sigma cat# E8263) and 0.5% sodium deoxycholate in a lysis buffer (150 mM NaCl, 50 mM Tris–HCl pH 8.4) for 30 min at 37 °C. Following incubation, the supernatant was supplemented with 5 M NaCl until a 1 M final concentration was achieved. Afterwards, the supernatant was lysed via 3 freeze thaw cycles at -80 °C and 50 °C. The lysate was spun down and the supernatant was transferred to an ultracentrifuge tube (Beckman cat# 342414), where it is layered with discontinuous layers of iodixanol (Accurate Chemical cat# AN1114542) to separate out viral particles from the supernatant. This was spun for 1 h at 18 °C at 69,000 rpm. The viral particles were isolated and removed, then washed four times in a dialysis column (Millipore cat# UFC910024) with PBS before being finally purified in a sterile filtration column (Millipore cat# UFC30DV00). Purified viruses were titered using quantitative PCR.

### Animals

The mice used in this study were described previously [34]. Briefly, the *Lrp1* floxed mice (*Lrp1*^*flox/flox*^) were bred with α-calcium-calmodulin–dependent kinase II (*CaMKII*)-driven *Cre* recombinase mice (*CaMKII-Cre*^+/-^) to generate the *Lrp1-*nKO mice (*Lrp1*^*flox/flox*^*; Cre*^+/-^*)* and the *Cre*-negative littermate controls (*Lrp1*^*flox/flox*^*; Cre*^*−/−*^*)*. The mice were maintained in the human *APOE3/3* background as described [34]. Animals were housed under controlled temperature and lighting conditions and were given free access to food and water. All animal procedures were approved by the Mayo Clinic Institutional Animal Care and Use Committee (IACUC) and were in accordance with the National Institutes of Health Guide for the Care and Use of Laboratory Animals.

### Stereotactic intracerebral AAV injection

For stereotaxic injection, adult *Lrp1*-nKO mice and control mice at 6 months of age were anesthetized with 2% isoflurane. Ibuprofen was given 48 h prior to surgery in drinking water. Using sterile instruments and gloves, a mid-sagittal longitudinal incision was made in the scalp to expose the skull, and a small burr hole was drilled through the skull with a hand drill to expose the brain. A 10 μL Hamilton syringe mounted on an electrode holder on the stereotaxic apparatus was inserted into the right hippocampus at the following coordinates: anterior posterior, − 2.5 mm; medial lateral, 1.5 mm; dorsal ventral, − 2.2 mm. The microinjections of AAVs (2 μL, 1.64 × 10^14^ viral genomes per mL) were performed at a rate of 0.5 μL per min and the needle was left in place for an additional 5 min after each injection. Following the injection, the needle was withdrawn, and the burr holes was covered with sterile Gelfoam® bone wax, with the purpose to seal the bone and prevent bleeding. The scalp was closed with surgical adhesive glue. One dose of Ampicillin was given (100 mg/kg) to prevent infection and one dose of Buprenorphine was given (0.05 mg/kg) to relieve pain. The animal was placed under a hot lamp until it regained its righting reflex and ambulated without problems, then placed in their cage. Mice received Ibuprofen in water for 5 days after surgery.

### Mouse brain tissue preparation

Three months after the AAV injection, mice were anesthetized and transcardially perfused with PBS. A 0.5 mm Mouse Brain Matrice (Alto) was used to coronally cut the brain tissues. Briefly, the olfactory bulb and cerebellum were removed, and the rest of the tissues were separated into two parts: the region of Bregma 3 mm to 1 mm was separated for biochemical analyses and the rest of tissues from Bregma 1 mm to -3 mm were fixed and used for immunofluorescent staining, according to the Allen Mouse Brain Atlas (See http://mouse.brain-map.org). For biochemical analysis, the brain tissues were homogenized and lysed in RIPA (Fisher Scientific) buffer, supplemented by protease inhibitor (cOmplete) and phosphatase inhibitor (PhosSTOP), and ultracentrifuged at 40,000 g for 20 min at 4 °C. The supernatant protein concentration was measured and normalized between samples. Samples were boiled in SDS loading buffer and used for Western blotting analysis. For immunofluorescent staining, the tissues were fixed in 4% paraformaldehyde at 4 °C for 48 h, and then washed in PBS and cryoprotected in PBS containing 30% sucrose. The tissues were then embedded in OCT containing 30% sucrose (at 1:1 v/v) for cryosections.

### Western blotting

The mouse brain tissues were prepared as mentioned above. The iPSNs were lysed in RIPA and the samples were prepared using the same protocol as mouse brain lysate. The prepared mouse brain and iPSN lysates were subjected to 4–20% SDS–PAGE (Bio-Rad) and transferred to polyvinylidene difluoride membranes, which were subsequently blocked using 5% milk in PBS. After blocking, proteins were detected with a primary antibody overnight at 4 °C. The next day, membranes were washed, and probed with horseradish peroxide (HRP)-conjugated secondary antibody and developed with enhanced chemiluminescence imaging. The primary antibodies were as follows: anti-human/mouse LRP1 (in-house antibody, clone 6F8, 1:1000), anti-β-actin (Sigma-Aldrich, cat# T2228, 1:3000).

### Immunofluorescent staining

The OCT-embedded brain tissues were cryosectioned at 40 µm thickness and the free-floating coronal brain sections were collected. The tissue was permeabilized with 0.25% Triton X-100 in PBS for 1 h, blocked in 10% goat serum for 1 h and incubated overnight with the primary antibodies of NeuN (Sigma-Aldrich, cat# MABN140, 1:500) and α-Syn (Biolegend, cat# 103–108, clone 4B12, 1:500). Sections were then incubated with Alexa Fluor-conjugated secondary antibodies for 2 h at room temperature. Fluorescent signals were detected by fluorescence microscopy (model BZ-X810, Keyence) or confocal laser scanning fluorescent microscopy (model LSM510 Invert, Carl Zeiss).

For the immunofluorescent staining with iPSNs, the cells were fixed in 4% paraformaldehyde and then permeabilized with 0.25% Triton X-100 in PBS. After blocking with 1% BSA in PBS for 30 min, cells were incubated with primary antibodies overnight at 4 °C. After washing with PBS, cells were incubated with Alexa Fluor-conjugated secondary antibodies for 2 h at room temperature. Fluorescent signals were detected by fluorescence microscopy (model BZ-X810, Keyence) or confocal laser scanning fluorescent microscopy (model LSM510 Invert, Carl Zeiss). The information of primary antibodies and their dilutions used in this study were as follows: Nanog (Cell Signaling, cat# 4903, 1: 300), TRA-1–60 (Abcam, cat# ab16288, 1: 300), Sox17 (Abcam, cat# ab84990, 1: 300), Brachyury (R&D, cat# AF2085, 1: 300), Nestin (Abcam, cat# ab18102, 1:300), and Tuj1 (Sigma-Aldrich, cat# T2200, 1:1000).

### Statistical analyses

All data were reported as mean ± s.d. All statistical analysis was performed using Prism 8 software. Unpaired t test was used for comparison between two groups and one-way analysis of variance (ANOVA) followed by Tukey’s multiple comparison test was used to compare outcomes among more than two groups. All statistical tests were two-sided. In the captions of the figures, we reported the used statistical tests for each analysis, the numerosity of the experiments, and the significance levels.

## Results

### Generation and characterization of the *LRP1*-KO iPSNs

**Fig. 1:**
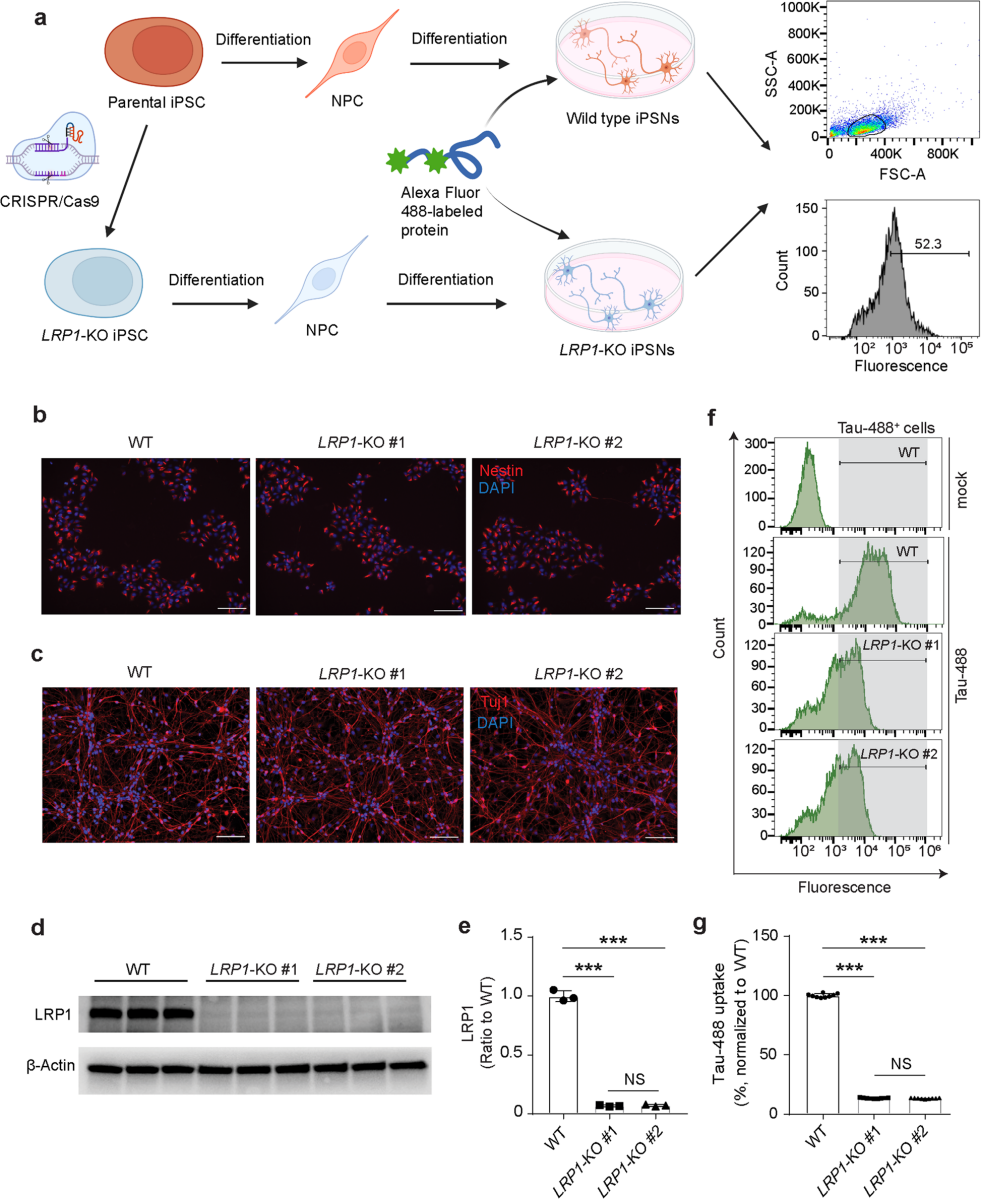
Generation and validation of human induced pluripotent stem cells (iPSCs)-derived neurons (iPSNs) with *LRP1* gene knockout (*LRP1*-KO). **a** Schematic diagram of the workflow for *LRP1*-KO iPSC generation, neural differentiation, and protein uptake assays. *LRP1*-KO iPSC colonies were obtained using CRISPR/Cas9 gene editing stratagy. Neural progenitor cells (NPCs) were then induced from the iPSCs and further differentiated into iPSNs. On day 14 to 16 of iPSN differentiation, neurons were treated with fluorescently labeled proteins and the uptake was measured by flow cytometry. **b** Immunofluorescence images showing NPCs from all three lines (WT, *LRP1*-KO#1, and *LRP1*-KO#2) were positive for neural precursor marker, Nestin. Scale bars, 100 μm. **c**, Immunofluorescence images of iPSNs from all three cell lines were positive for neuronal marker, Tuj1. Scale bars, 100 μm. **d** and **e**, Detection and quantification of LRP1 protein levels in WT and *LRP1*-KO iPSNs via Western blotting. **f** and **g**, Endocytosis of human tau in WT and *LRP1*-KO iPSNs measured by flow cytometry (100 nM, 3 h of treatment). Experiments in (**f** and **g**) were performed in technical duplicates or triplicates over three independent experiments. All data are expressed as mean ± s.d. with individual data points shown. Data were analyzed by One-way ANOVA with Tukey’s multiple comparisons test. NS, not significant; ***P < 0.001

To explore the role of LPR1 in α-Syn uptake, we generated *LRP1*-KO human iPSCs using the CRISPR/Cas9 technique. Two *LRP1*-KO iPSC clones were obtained and the *LRP1* deletion was validated via Sanger DNA sequencing in each clone (Fig. S1b). Karyotyping in each iPSC clones revealed the preservation of normal number and appearance of chromosomes (Fig. S2a). We further confirmed that the *LRP1*-KO iPSCs and their parental wild type (WT) iPSCs expressed pluripotent stem cell-specific markers, including Nanog and TRA-1–60 (Fig. S2b). The parental and isogenic iPSC lines were then differentiated into NPCs (Fig. 1a) and we confirmed that all the NPCs from the three lines were positive for neural precursor marker (Nestin) by immunostaining (Fig. 1b). We then differentiated the NPCs into neurons by culturing the cells in neuronal differentiation medium (Fig. 1a). After 14 days of culture, both WT and *LRP1*-KO cells exhibited a typical neuronal morphology and expressed the neuronal marker (Tuj1), suggesting the successful differentiation of NPCs into neurons (iPSNs) (Fig. 1c). We then confirmed the deletion of LRP1 in these iPSNs by Western blotting analysis (Fig. 1d and e).

To validate the previously reported role of LRP1 in tau endocytosis using our *LRP1*-KO iPSNs [29], we treated the cells with 100 nM fluorescently labeled tau proteins. Three hours after the tau treatment, we harvested the iPSNs and measured the endocytosis of tau proteins by flow cytometry. We confirmed that the amount of internalized tau proteins was reduced by ~ 90% in *LRP1*-KO iPSNs compared to WT cells (Fig. 1f and g), consistent with what has been reported previously. Together, these data indicate that our strategy of deleting LRP1 in human iPSNs was successful and tau uptake was inhibited by LRP1 deletion in these cells.

### LRP1 regulates α-Syn uptake in iPSNs

**Fig. 2:**
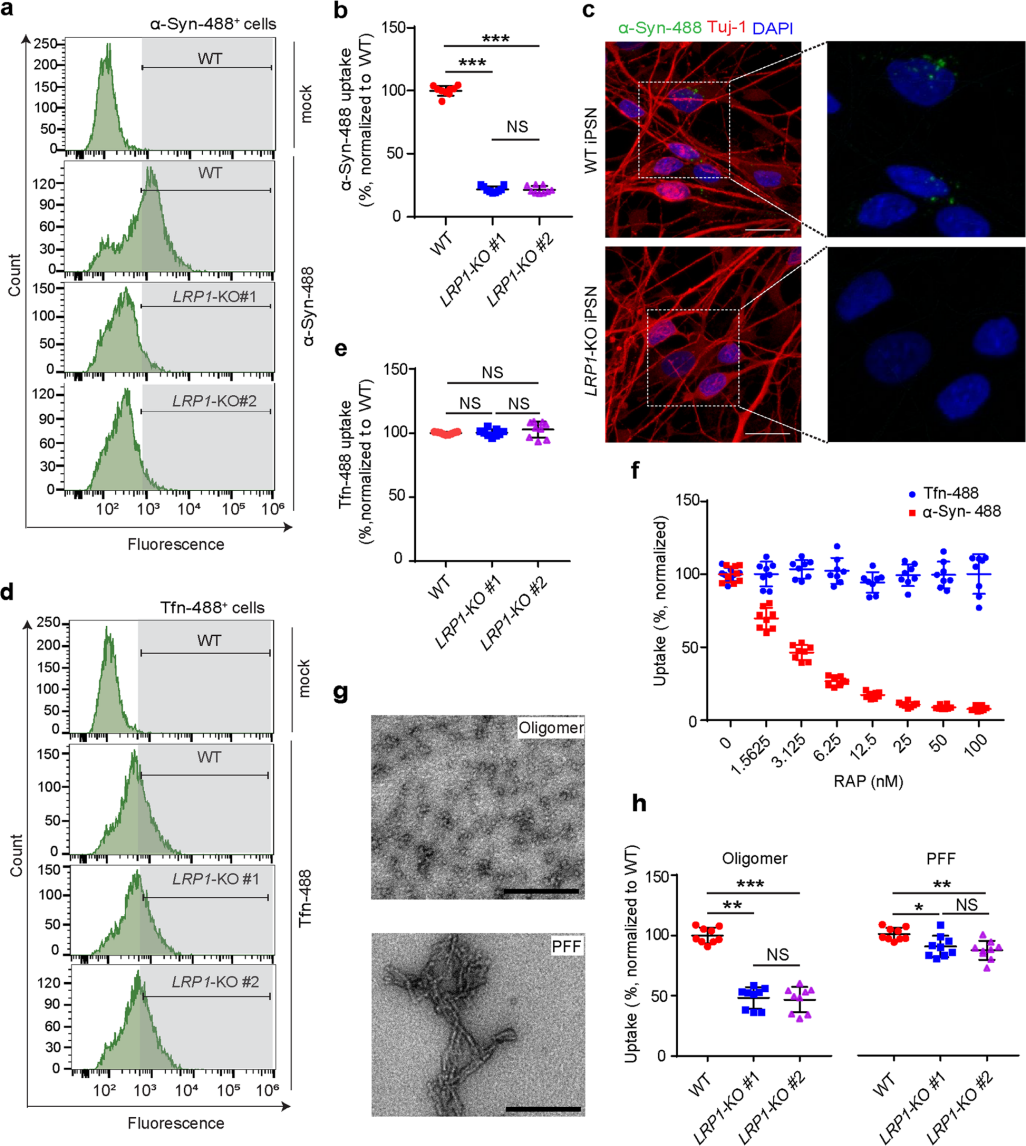
LRP1 regulates α-Syn uptake in iPSNs. **a** and **b**, α-Syn uptake in WT and *LRP1*-KO iPSNs measured by flow cytometry (100 nM, 3 h of treatment). **c** Representative images of WT or *LRP1*-KO iPSNs after α-Syn uptake. Scale bars, 20 μm. **d** and **e**, Transferrin (Tfn) uptake in WT and *LRP1*-KO iPSNs measured by flow cytometry (300 nM, 3 h of treatment). **f** Uptake of α-Syn and Tfn in the presence of increasing concentrations of RAP. **g** EM images showing the structure of α-Syn oligomers and preformed fibrils (PFFs) used in panel h. Scale bars, 200 nm. **h** Uptake of α-Syn oligomers and PFFs in WT and *LRP1*-KO iPSNs (100 nM monomer equivalent, 3 h of treatment). All experiments in (**a**, **b**, **d**,** e**,** f** and **h**) were performed in technical duplicates or triplicates over three independent experiments. All data are expressed as mean ± s.d. with individual data points shown. Data were analyzed by One-way ANOVA with Tukey’s multiple comparisons test. NS, not significant; *P < 0.05, **P < 0.01, ***P < 0.001

To test whether the uptake of α-Syn is through a similar mechanism as tau in neurons, we treated the iPSNs with fluorescently labeled monomeric α-Syn and measured the cellular α-Syn signal by flow cytometry after 3 h of treatment at a concentration of 100 nM. We found that, similar to tau, LRP1-deficiency significantly reduced the cellular uptake of α-Syn (more than 75% reduction) in the iPSNs and consistent findings were observed from two different clones of *LRP1*-KO iPSNs (Fig. 2a, b, and c). To further confirm that *LRP1*-KO specifically impacts α-Syn and tau internalization, we treated the iPSNs with fluorescently labeled transferrin and we did not observe any reduction of transferrin uptake in the *LRP1*-KO iPSNs, as expected (Fig. 2d, e). Next, we tested whether the RAP, a known LRP1 binding antagonist [35], could compete with the α-Syn for neuronal uptake by LRP1. We treated the iPSNs with 100 nM of α-Syn together with different concentrations of RAP ranging from 1.5625 nM to 100 nM. We found that RAP strongly suppressed the neuronal uptake of α-Syn in a dose-dependent manner, whereas transferrin internalization was not affected (Fig. 2f). Together, these results suggest that LRP1 is a key endocytic receptor for monomeric α-Syn in neurons.

Additionally, we tested if LRP1 also mediates oligomeric and fibrillar α-Syn uptake. We treated the iPSNs with 100 nM (monomer equivalent) of fluorescently labeled α-Syn oligomers and sonicated α-Syn PFFs. The structure of α-Syn oligomers and PFFs were confirmed under electron microscopy (EM) (Fig. 2g). We found that *LRP1*-KO also effectively inhibited the uptake of α-Syn oligomers (~ 50% reduction) (Fig. 2h). However, unlike tau fibrils [29], *LRP1*-KO only had a mild inhibitory effect on the uptake of α-Syn PFFs (~ 5–10% reduction) (Fig. 2h). These findings indicate that LRP1 may be the primary receptor for the uptake of soluble α-Syn, including monomer and oligomer; and the internalization of aggregated α-Syn may be mediated through a different or multiple mechanisms.

### LRP1 regulates the uptake of α-Syn via lysine residues in the N-terminus of α-Syn

**Fig. 3:**
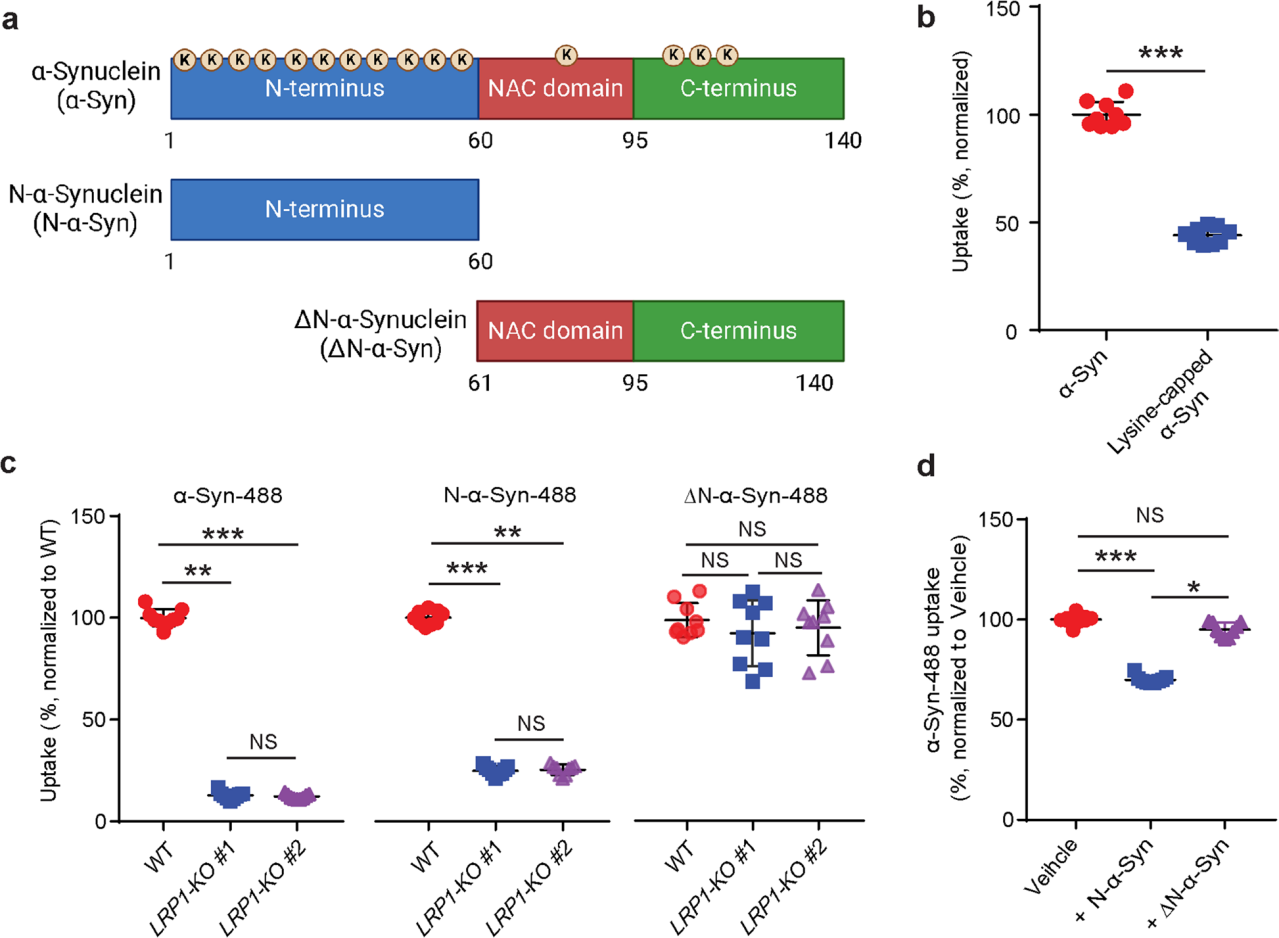
LRP1 regulates α-Syn uptake via lysine residues in the N-terminus of α-Syn. **a** Schematic diagram of α-Syn domains highlighting the lysine residues (K). **b** Uptake of α-Syn and lysine-capped α-Syn in WT iPSNs. **c**, Uptake of α-Syn-488, N-α-Syn-488 and ΔN-α-Syn-488 in WT and *LRP1*-KO iPSNs. **d** Uptake of α-Syn-488 in the presence of excessive non-labeled α-Syn N-terminus (N-α-Syn) or α-Syn lacking N-terminus (ΔN-α-Syn) fragments. All experiments in (**b**–**d**) were performed in technical duplicates or triplicates over three independent experiments. All data are expressed as mean ± s.d. with individual data points shown. Data in (**b**) was analyzed by unpaired two-sided t-test. ***P < 0.001. Data in (**c** and **d**) were analyzed by One-way ANOVA with Tukey’s multiple comparisons test. NS, not significant; *P < 0.05, **P < 0.01, ***P < 0.001

LRP1 contains ligand-binding domains with cysteine-rich complement-type repeats [36]. The aspartic acid residues in each repeat can form acidic pocket responsible for docking the lysine residues on ligands [37]. Monomeric α-Syn is composed of three domains: a lipid binding N-terminus domain (aa 1–60), a hydrophobic non-amyloid component (NAC) domain (aa 61–95), and a negatively charged C-terminus domain (aa 96–140) [2]. α-Syn has a high lysine content (15 lysines out of 140 aa, ~ 10%) with most (eleven) lysines located in the N-terminus, one lysine in NAC, and three in C-terminus (Fig. 3a). To examine whether the lysine residues of α-Syn were involved in LRP1-medicated α-Syn uptake, we capped the amines of lysines on α-Syn with sulfo-NHS acetate and then measured the internalization of α-Syn in the iPSNs. We found that the blocking of lysine residues on α-Syn effectively decreased the uptake of α-Syn in the iPSNs (~ 50% reduction) (Fig. 3b), indicating that lysine residues of α-Syn are critical for LRP1-mediated α-Syn uptake.

We next sought to determine whether LRP1 regulates α-Syn uptake through the recognition of the lysine-rich N-terminus of α-Syn. We employed the α-Syn protein fragments containing only the N-terminus (N-α-Syn) or lacking the N-terminus (ΔN-α-Syn) of full-length α-Syn (Fig. 3a). We then labeled the N-α-Syn and ΔN-α-Syn fragments with fluorescence and examined their endocytosis in iPSNs. We found that *LRP1*-KO effectively inhibited the uptake of N-α-Syn, to a similar extent as the full-length α-Syn but had no effect on ΔN-α-Syn uptake (Fig. 3c). To further support this finding, we tested whether N-α-Syn or ΔN-α-Syn protein fragment could compete for the uptake of the full-length α-Syn. We treated the iPSNs with fluorescently labeled full-length α-Syn (100 nM), and the five-fold molar excess of non-labeled N-α-Syn or ΔN-α-Syn fragments (500 nM). After 3 h of treatment, we found that the addition of N-α-Syn significantly inhibited the uptake of full-length α-Syn, whereas ΔN-α-Syn had no effect (Fig. 3d). Taken together, these data indicate that LRP1 regulates the uptake α-Syn via lysine residues and N-terminus of α-Syn is critical for LRP1-mediated α-Syn uptake.

### Neuronal Lrp1 deletion reduces α-Syn spread in mouse model

**Fig. 4:**
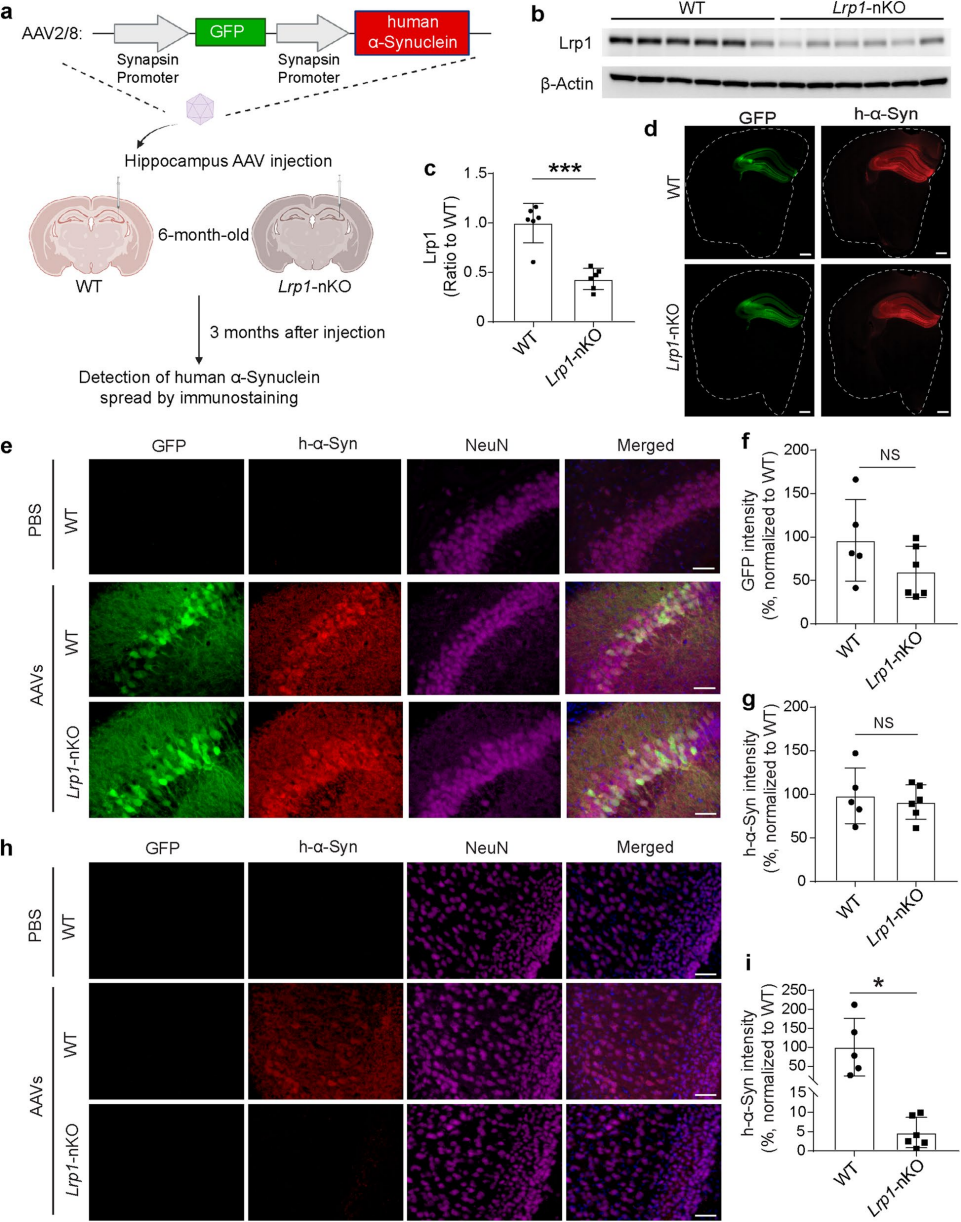
Neuronal *Lrp1* knockout reduces α-Syn spread in vivo. **a** Schematic drawing for the stereotactic injection of *AAV-synapsin-GFP-synapsin-h-α-Synuclein* into the neuronal *Lrp1* knockout (*Lrp1*-nKO) mice and wild type (WT) littermate controls, and the experimental workflow. **b** and **c**, Western blotting showing the endogenous Lrp1 protein in the cortex of WT (*n* = 6) and *Lrp1*-nKO mice (*n* = 6). **d** Representative sections showing GFP and h-α-Syn signals in mouse brains. Dotted line marks the outline of each section. Scale bars, 500 μm. **e** Representative images showing GFP and h-α-Syn signals in the hippocampus region from WT and *Lrp1*-nKO mice. Scale bars, 50 μm. **f** Quantitative analysis of GFP intensity in hippocampus from WT or *Lrp1*-nKO mice. **g** Quantitative analysis of h-α-Syn immunofluorescence intensity in hippocampus from WT or *Lrp1*-nKO mice. **h** Representative images showing h-α-Syn spreading to the cortex region from WT or *Lrp1*-nKO mice. Scale bars, 50 μm. **i** Quantitative analysis of h-α-Syn immunofluorescence intensity in the cortex from WT or *Lrp1*-nKO mice. Experiments in (**e**–**i**) *n* = 5 mice (3 males and 2 females) for WT and *n* = 6 mice (3 males and 3 females) for *Lrp1*-nKO mice. All data are expressed as mean ± s.d. with individual data points shown. Data were analyzed by unpaired two-sided t-test. NS, not significant; *P < 0.05, ***P < 0.001

Next, we tested whether Lrp1 was also critical for the spread of α-Syn in neurons in vivo using a mouse model. We utilized an AAV approach that allowed us to visualize the α-Syn spread between hippocampus and cortex. The AAV contained dual synapsin promoters that allowed efficient co-expression of GFP and h-α-Syn in neurons (*AAV-synapsin-GFP-synapsin-h-α-Synuclein*) (Fig. 4a). Neurons that were transduced with the virus could produce GFP and h-α-Syn as two separate proteins; whereas neurons that received h-α-Syn through cell-to-cell spreading would have only h-α-Syn but not GFP protein based on the design of the AAV construct (Fig. 4a). To investigate the role of Lrp1 in α-Syn spread in animals, we used the conditional neuronal *Lrp1* knockout mice [38] by crossing *Lrp1*^*flox/flox*^ mice with *CaMKII*-*Cre* mice to generate *Lrp1*-nKO (*Lrp1*^*flox/flox*^*; Cre*^+*/−*^) and WT littermate controls (*Lrp1*^*flox/flox*^*; Cre*^*−/−*^). It has been reported that the deletion of *Lrp1* driven by *CaMKII*-*Cre* was significant in the cortex and hippocampus after 6 months of age [39]. Therefore, we performed stereotactic injection of the AAVs into the right hippocampus of these mice at 6 months of age. Three months after the injection, the mice were euthanized for the evaluation of the h-α-Syn spreading (Fig. 4a). We first confirmed that endogenous Lrp1 protein level was significantly reduced in the *Lrp1*-nKO mice compared to WT controls measured by Western blotting (Fig. 4b, c). We observed that the GFP signal was exclusively expressed in the hippocampus but not in other brain regions (Fig. 4d), suggesting that only the neurons in this region were transduced by the AAVs as designed (Fig. 4e). Within the hippocampus, both GFP and h-α-Syn signal showed no significant differences between *Lrp1*-nKO and WT mice, indicating equivalent transduction of AAVs between the two groups (Fig. 4e, f and g). Interestingly, in the cortex above hippocampus, we found neurons that had only h-α-Syn but not GFP signals, suggesting that these neurons received h-α-Syn from hippocampus. The quantification of these h-α-Syn signals in the cortex revealed significant reduction in the *Lrp1*-nKO mice compared to WT controls (Fig. 4h, i), indicating that Lrp1 deletion suppresses the spread of α-Syn in mouse brain.

## Discussion

In this study, we investigated the role of LRP1 in α-Syn uptake and spread. We found that LRP1-deficiency significantly reduced neuronal α-Syn and tau uptake using *LRP1*-KO iPSNs generated by the CRISPR/Cas9 technique. Importantly, we also confirmed that LRP1 mediates α-Syn spread in mouse brains using a transgenic mouse model deleting the *Lrp1* gene in neurons. Our findings support the hypothesis that LRP1 is a key regulator for both α-Syn and tau uptake and spread, providing potential therapeutic insight targeting the relevant diseases with α-Syn and tau pathogenesis.

The spread of conformationally distinct pathological protein aggregates between anatomically connected brain regions is a common feature of multiple neurodegenerative diseases, including tauopathies and synucleinopathies [30, 40]. Various molecular mechanisms related to the transmission of tau and α-Syn pathology have been reported, including the receptor-mediated endocytosis [29, 41, 42], exosomal transport [43–45], tunneling nanotubes transport [46–48], etc. Among these distinct mechanisms, the LRP1-mediated cellular uptake and spread is shown as a common mechanism for the cell-to-cell transmission of both tau [29, 42] and α-Syn (from this study). As a cell surface receptor, LRP1 regulates the endocytosis of a long list of ligands through direct interaction. We showed that LRP1 interacts with the N-terminus of α-Syn through lysine residues, similar to how LRP1 interacts with tau [29]. We observed that neuronal LRP1 is a key regulator for the endocytosis of monomeric and oligomeric α-Syn. However, different from tau [29], our study shows that the uptake of fibrillar form of α-Syn is less dependent on LRP1 in neurons. It has been reported that in the disease-associated, aggregated state of α-Syn, the N-terminal residues (37 through 97), adopts a β-sheet structure [49]. The finding may suggest that when forming the fibrils, the N-terminus of α-Syn is less exposed therefore the interaction between α-Syn and LRP1 might be limited. A recent study showed that the lymphocyte-activation gene 3 (LAG3) is a receptor for α-Syn PFFs which mediates the endocytosis and transmission of α-Syn PFFs between neurons [41]. Notably, LAG3 specifically binds to α-Syn PFFs but not monomers. These findings raise the possibility that different forms of α-Syn might be recognized by distinct receptors. Although the role of the uptake and spread of soluble α-Syn monomers is still poorly understood, it is well-documented that the α-Syn oligomers are neurotoxic and are the major α-Syn species serving as the “seeds”, leading to the α-Syn aggregates [12–14, 50, 51]. Therefore, future studies could focus on identifying therapeutic strategies to disrupt the uptake and spread of oligomeric α-Syn species via LRP1-related pathway.

LRP1 is widely expressed in a variety of cell types including neurons, astrocytes, microglia, macrophages, fibroblasts, and smooth muscle cells. In our study, we demonstrated the role of neuronal LRP1 in mediating tau and α-Syn uptake in neurons. However, whether other cell types, such as astrocyte and microglia, can also take up and spread tau and α-Syn via LRP1-mediated mechanism needs further investigation.

The ε4 allele of the *APOE* gene (*APOE4*) is a strong genetic risk factor for Lewy body dementia (LBD) [52, 53]. *APOE4* has also been implicated in the progression of cognitive impairment or motor dysfunction within PD [54–61]. Recently, we reported that the presence of *APOE4* gene allele exacerbates α-Syn seeding in a large AD cohort and a small cohort of LBD cases [62]. These findings indicate that APOE4 may potentially accelerate α-Syn aggregation and spreading during the disease [63]. In fact, LRP1 is the major metabolic receptor for APOE in the brain [25]. LRP1 mediates the transport of cholesterol and phospholipids into CNS neurons through binding with APOE to support synaptic integrity and plasticity. Therefore, it is possible that APOE4 affects α-Syn aggregation and spreading via LRP1-related mechanisms. Additionally, heparan sulfate proteoglycans (HSPGs), another APOE receptor, have also been shown to be involved in both tau and α-Syn endocytosis [64, 65]. It has been reported that HSPGs bind to α-Syn fibrils and facilitate their endocytosis [64]. LRP1 is known to work in conjunction with HSPGs. For example, LRP1 and HSPGs cooperatively mediate cellular Aβ uptake [66]. How LRP1, HSPGs, and APOE together affect the uptake and spread of tau and α-Syn needs to be further investigated in proper model systems.

In summary, our study defines LPR1 as a key receptor for α-Syn uptake and spread in neurons, presenting a potential therapeutic target for the treatment of synucleinopathies.

## Supplementary Information

**Additional file 1: Fig. S1.** Generation of *LRP1*-KO iPSC lines. **a** gRNAs are designed to target exon 6 of human *LRP1* gene. **b** Sequencing results of *LRP1*-KO iPSC clones. Both *LRP1*-KO #1 and *LRP1*-KO #2 clones exhibit a deletion of 191 bp of exon 6, causing a frameshift and a premature stop codon. **Fig. S2.** Characterization of parental and *LRP1*-KO iPSCs. **a** Karyotyping for the iPSCs. **b** Immunostaining for pluripotency markers (Nanog and TRA-1- 60). Scale bars, 100 μm.

## Data Availability

The data of this study are available from the corresponding author on reasonable request.

## Abbreviations

α-Syn: α-Synuclein
PD: Parkinson’s disease
KO: Knockout
iPSCs: Induced pluripotent stem cells
iPSNs: Induced pluripotent stem cell-derived neurons
PFFs: Pre-formed fibrils
N-α-Syn: α-Syn N-terminus
ΔN-α-Syn: α-Syn lacking N-terminus
AAV: Adeno-associated virus
SNARE: Soluble N-ethylmaleimide–sensitive factor attachment protein receptor
ISF: Interstitial fluid
CSF: Cerebrospinal fluid
LRP1: Low-density lipoprotein receptor-related protein 1
LDLR: Low-density lipoprotein receptor
APOE: Apolipoprotein E
Aβ: Amyloid-beta
AD: Alzheimer's disease
NPCs: Neural progenitor cells
RAP: Receptor-associated protein
EM: Electron microscopy
NAC: Hydrophobic non-amyloid component
LAG3: Lymphocyte-activation gene 3
LBD: Lewy body dementia
HSPGs: Heparan sulfate proteoglycans
