# Supplementary material for: LRP1 is a neuronal receptor for α-synuclein uptake and spread
Source: Mol Neurodegener. 2022 Sep 2;17:57. doi: 10.1186/s13024-022-00560-w (PMC9438229; doi:10.1186/s13024-022-00560-w)
Supplement: Supplementary file 1 — Additional file 1: Fig. S1. Generation of LRP1-KO iPSC lines. a gRNAs are designed to target exon 6 of human LRP1 gene. b Sequencing results of LRP1-KO iPSC clones. Both LRP1-KO #1 and LRP1-KO #2 clones exhibit a deletion of 191 bp of exon 6, causing a frameshift and a premature stop codon. Fig. S2. Characterization of parental and LRP1-KO iPSCs. a Karyotyping for the iPSCs. b Immunostaining for pluripotency markers (Nanog and TRA-1- 60). Scale bars, 100 μm. [file 13024_2022_560_MOESM1_ESM.pdf]

a

b

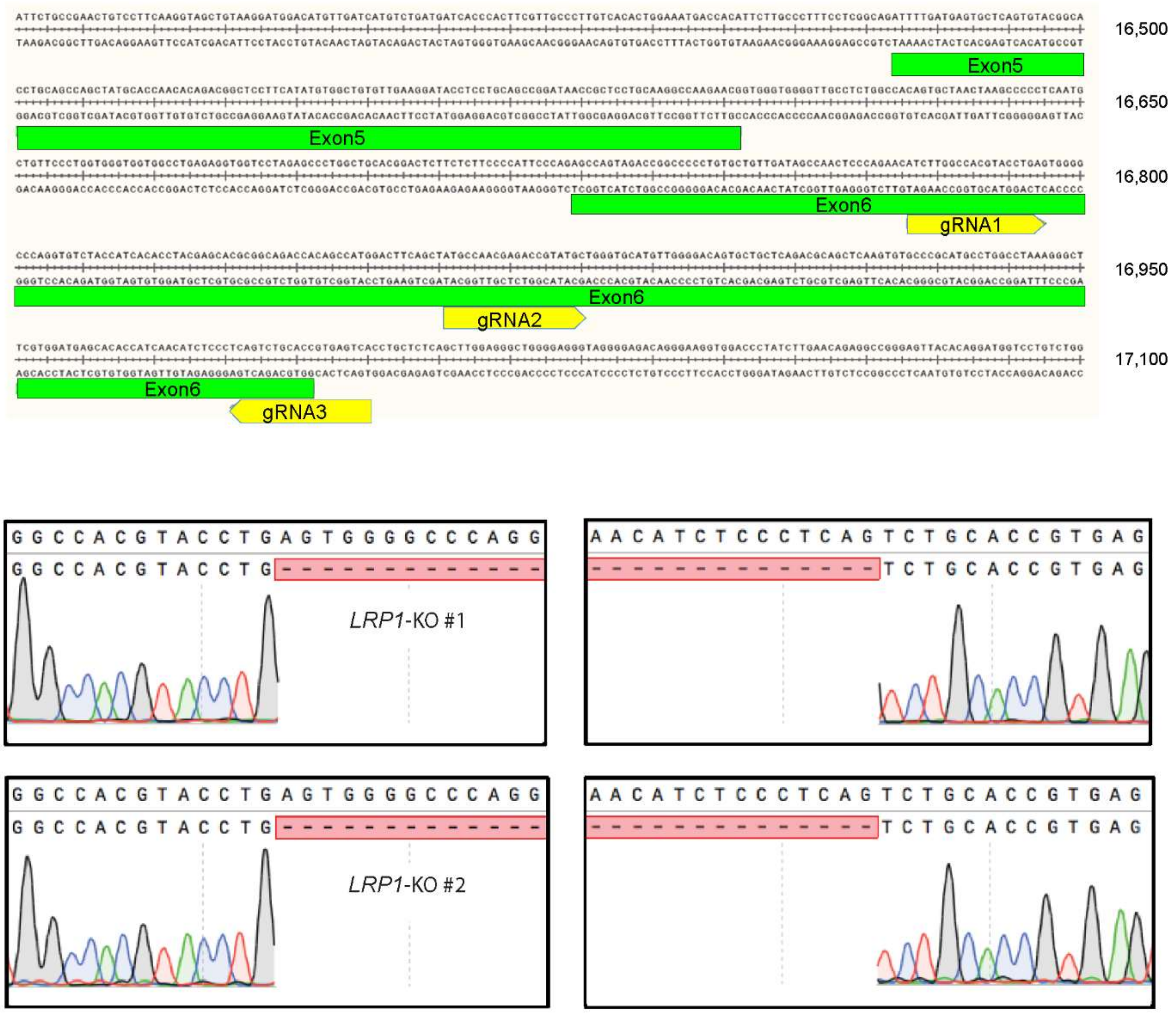


**Fig. S1 Generation of *LRP1*-KO iPSC lines**

**a,** gRNAs are designed to target exon 6 of human *LRP1* gene. **b**, Sequencing results of *LRP1*-KO iPSC clones. Both *LRP1*-KO #1 and *LRP1*-KO #2 clones exhibit a deletion of 191 bp of exon 6, causing a frameshift and a premature stop codon.

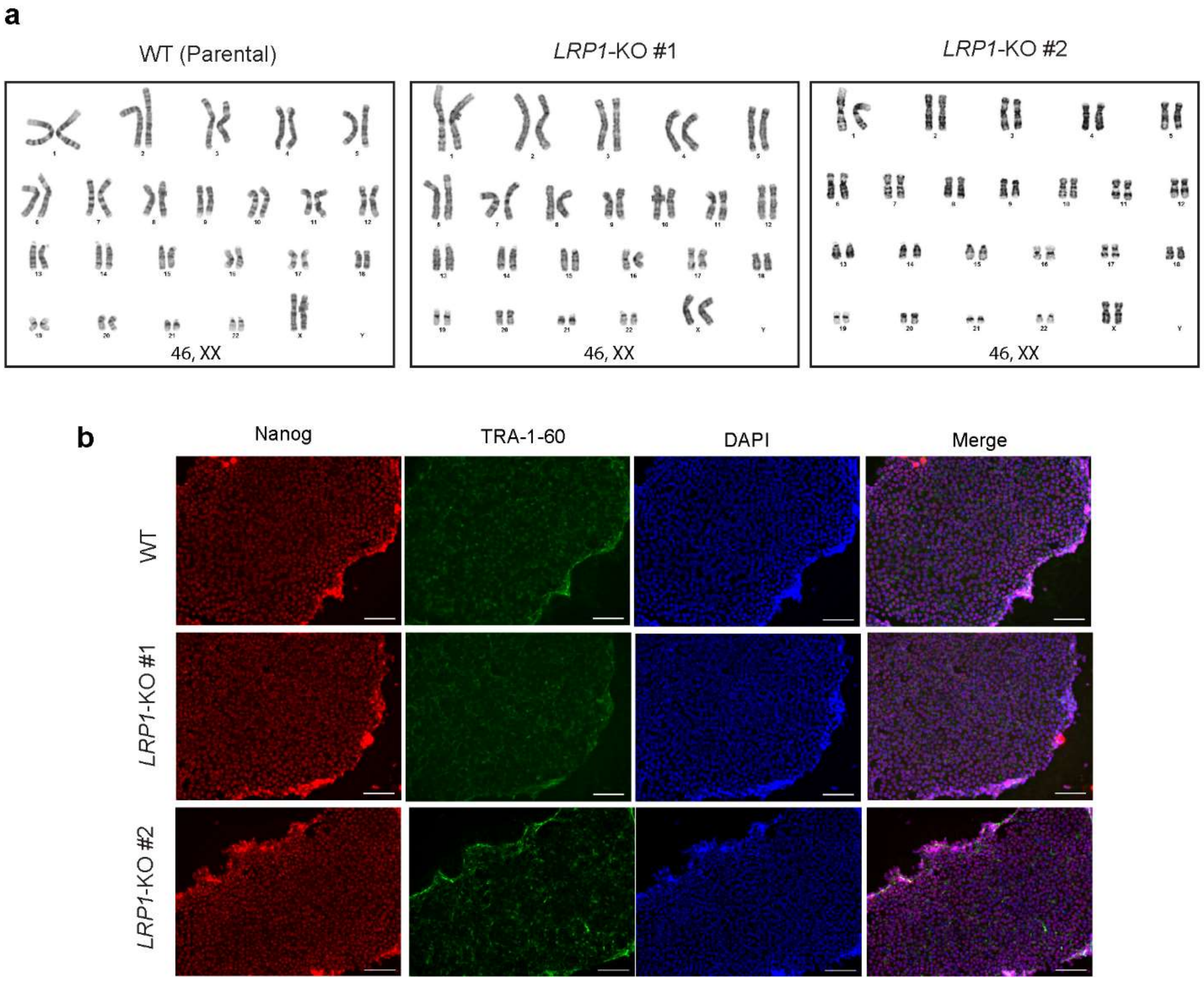


**Fig. S2 Characterization of parental and *LRP1*-KO iPSCs**

**a,** Karyotyping for the iPSCs. **b**, Immunostaining for pluripotency markers (Nanog and TRA-1-60). Scale bars, 100 µm.
